# Low Levels of Usual Physical Activity Are Associated with Higher 24 h Blood Pressure in Type 2 Diabetes Mellitus in a Cross-Sectional Study

**DOI:** 10.1155/2017/6232674

**Published:** 2017-09-06

**Authors:** Alessandra Teixeira Neto Zucatti, Tatiana Pedroso de Paula, Luciana Verçoza Viana, Rafael DallAgnol, Felipe Vogt Cureau, Mirela Jobim Azevedo, Jorge Luiz Gross, Beatriz D. Schaan, Cristiane Bauermann Leitao

**Affiliations:** Endocrine Division, Hospital de Clínicas de Porto Alegre, Universidade Federal do Rio Grande do Sul, Porto Alegre, RS, Brazil

## Abstract

The aim of this study is to evaluate the association between usual physical activity and 24 h blood pressure (BP) profile in people with type 2 diabetes mellitus (DM). This is a cross-sectional study of 151 participants with type 2 DM. Usual physical activity was assessed by step counting and self-reported questionnaire. BP was measured in office and by 24 h ambulatory BP monitoring (ABPM; 24 h, daytime and nighttime). Mean participant age was 61.1 ± 8.4 years, 64% was women, and mean duration of diabetes was 14.3 ± 8.5 years. Ninety-two percent of participants had hypertension, and office BP was 138 ± 18/78 ± 10 mmHg. Inverse correlations were observed between step count and 24 h BP (systolic, *r* = −0.186; *p* = 0.022), daytime BP (systolic, *r* = −0.198; *p* = 0.015), and nighttime BP (pulse pressure, *r* = −0.190; *p* = 0.019). People were categorized into tertiles of daily step count, and the 1st tertile had higher 24 h systolic BP, daytime systolic BP, daytime mean BP, and daytime systolic BP load than those in the other tertiles, even after adjusting for age and HbA1c. Participants with type 2 DM and low levels of physical activity exhibit higher 24 h and daytime systolic ambulatory BP values as compared with those who performed more steps per day, even after adjustments for confounding factors.

## 1. Introduction

Hypertension is a major risk factor for the development and progression of chronic complications in diabetes mellitus. Sixty to 80% of persons with type 2 DM have hypertension [1, 2], and the majority of these have blood pressure (BP) values above the recommended targets even after intensive optimization of antihypertensive therapy [3, 4]. Lifestyle changes and antihypertensive medications are the cornerstone of treatment for these persons [1, 3, 5], and exercise is an important part of the diabetes management plan [3].

Current guidelines recommend that everyone who has no contraindications should engage in regular aerobic physical activity (at least 150 minutes per week of moderate to intense physical activity, or 50–70% of maximum heart rate), but this suggestion is mainly based on benefits found in trials evaluating glucose control [3, 6]. All types of structured exercise, including aerobic, resistance, and their combination, are associated with improvement in glucose control in type 2 DM [7], but the precise effect of these different modalities of exercise on BP control in persons with diabetes has been questioned. A previous meta-analysis showed a significant reduction in systolic BP associated with aerobic training and combined exercise, but no effect was observed for resistance training [8]. Recently, we evaluated the effects of structured exercise training and physical activity advice only on BP of people with type 2 DM [9]. In our meta-analysis, aerobic and resistance exercises were associated with declines in BP, but only a high-intensity protocol of combined training was able to impact BP.

However, a structured exercise program is not available to all patients with diabetes, and most of them will simply be advised to increase physical activity by their primary care physician. A home-based gradual physical activity program, designed to achieve a goal of 175 min of moderate intensity physical activity per week, was evaluated with other lifestyle changes as a cointervention [10] and found to reduce BP (systolic: −4.0 mmHg; diastolic: −1.2 mmHg). We also have analyzed studies evaluating the effect of physical activity advice only on BP [9], and this simpler intervention was associated with reduction in systolic (−2.97 mmHg) and diastolic BP (−1.41 mmHg).

Low cardiorespiratory fitness, which is a hallmark of sedentary behavior, is a strong risk factor for mortality among males with diabetes [11]. Moreover, increased physical activity, including regular walking, is associated with substantially reduced risk for cardiovascular events in women with diabetes [12]. The number of steps walked per day, measured by a pedometer, is associated with a better cardiovascular risk profile in women [13]. In persons with type 2 DM, the number of daily steps is associated with a lower body mass index (BMI) [14], higher aerobic capacity evaluated by maximal oxygen consumption [15], better glycemic control [16], and arterial stiffness [17]. Daily steps and office BP values were evaluated in people with type 2 DM, which showed that each increment of 1000 steps/day was associated with a decrease of 2.6 mmHg on systolic and a 1.4 mmHg on diastolic BP, although only in women [18]. Traditionally, the pedometer-determined physical activity cutoff points for classification of healthy adults are <5000 steps/day (sedentary), 5000–7499 steps/day (low active), 7500–9999 steps/day (somewhat active), 10,000–12,499 (active), and ≥12,500 (highly active) [19], and 10,000 steps/day are suggested as a reasonable target for healthy adults [20]. However, considering people with diabetes, this goal may be not feasible and lower step counts may be associated with benefits, as it has been demonstrated for sedentary adults and older adults [21].

As 24 h BP profiles correlate better with outcomes than office BP, particularly in people with diabetes [22, 23], the present study sought to evaluate if low levels of usual physical activity (step count as measured by a pedometer) are associated with higher BP (measured by ambulatory BP monitoring [ABPM]) in persons with type 2 DM.

## 2. Material and Methods

### 2.1. Participants

This was a cross-sectional study of 151 participants with type 2 DM recruited from the outpatient clinics of a tertiary center. The inclusion criterion was a diagnosis of type 2 DM (age > 30 years at onset of diabetes, no previous episodes of ketoacidosis or documented ketonuria). Persons with creatinine levels > 2.0 mg/dL, any disease that interferes with glucose control (infection, prolonged use of corticosteroids), unstable angina or acute myocardial infarction in the last 3 months, NYHA class III or IV heart failure, cirrhosis, alcoholism or illegal drug use, dementia, pregnancy or lactation, malignancies, BMI > 40 kg/m^2^, physical disability preventing use of a pedometer, or participation in other research projects involving any type of intervention were excluded. The study protocol was previously approved by the Institutional Research Ethics Committee, and written informed consent was obtained from all participants.

### 2.2. Study Design

Participants underwent an interview and clinical examination for collection of demographic and anthropometric data. Blood pressure was measured in office and by 24 h ABPM. The mean of two office BP measurements, determined in two separate days (one in the same day of ABPM placement) and obtained with a digital sphygmomanometer (Omron HEM-705CP Blood Pressure Monitor) on the left arm and with the person in the seated position, after a 5-minute rest, was considered for all analyses. Ambulatory BP monitoring was performed by the oscillometric method (Spacelabs 90207, serial numbers 207-054280, 207-024751, 207-054290, 207-056568, and 207-038016, with calibration certification), with a 15-minute interval in the daytime and a 20-minute interval in nighttime periods. Participants were advised to maintain their usual daily activities. Sleep time was recorded as the period between the time when a participant went to bed and the time when the participant woke up in the morning. All ABPM measurements were obtained on a normal workday. The means of 24 h and daytime and nighttime systolic and diastolic BP were recorded, as well as BP loads (percentage of 24 h and daytime BP measurements ≥ 140/90 mmHg and nighttime BP measurements ≥ 120/80 mmHg). Hypertension was defined by the mean of office BP measurement ≥ 140/90 mmHg on two occasions or use of antihypertensive medications.

Usual physical activity was measured objectively by step counting with a pedometer (Digi-walker SW-700, Yamax Corporation, Tokyo, Japan). Participants wore pedometers for 7 days, attached to the waistband of their clothing during waking hours, except when bathing or swimming. Participants were encouraged not to alter their usual physical habits during these 7 days. Every evening, participants recorded their number of daily steps in a diary and the pedometer was reset to zero to be worn on the next day. The pedometer also provides data on the daily distance walked in kilometers.

Physical activity was also evaluated subjectively by self-reported levels of overall physical activity with the validated Portuguese-language version of the International Physical Activity Questionnaire (IPAQ long form) [24], and participants were classified into active (≥150 minutes of moderate to vigorous physical activity in the last week) or inactive (<150 minutes/week) [3]. Participants were also asked to complete a daily physical activity log, in which they recorded the type and duration (in minutes) of any sport or exercise (planned physical activity) performed during the week. Mean steps walked per day and minutes of planned physical activity per day were then computed for each participant. Visit plans were visit 1 (day 1)—participants underwent an interview and clinical examination for collection of demographic and anthropometric data; physical activity was evaluated by IPAQ. Office BP was measured and the pedometer delivered. Patients were advised to initiate the pedometer use in the next morning. A fasting blood sample was obtained, office BP was measured, and an ABPM device was placed—visit 2 (day 8). This was the last day of pedometer registration. The ABPM device was removed, and pedometers were returned to research center—visit 3 (day 9).

### 2.3. Laboratory Methods

Fasting plasma glucose levels were measured by the hexokinase method, and HbA1c was measured by a high-performance liquid chromatography (HPLC) assay (Merck-Hitachi 9100, normal range 4–6%). Total cholesterol, HDL cholesterol, and triglycerides were measured by the colorimetric method, and LDL was calculated with the Friedewald formula. Creatinine was measured by the Jaffé method. Albuminuria was measured in a random sample, and a value greater than 17 mg/L was considered diabetic renal disease.

### 2.4. Statistical Analysis

Data are expressed as mean (±SD), median (interquartile range), or absolute and relative frequencies. Variable distributions were evaluated by a Kolmogorov-Smirnov test. Pearson correlation coefficients were computed between the number of steps/day and the BP variables. One-way ANOVA (Bonferroni as post hoc test) or Kruskal-Wallis test (Bonferroni post hoc corrected alpha < 0.001) was used according to the data distribution, and the chi-square test was used for comparison of categorical variables. Differences in office BP and ABPM values among categories of physical activity were evaluated. The sample was divided in tertiles of step count per day to better explore the association between this variable and BP measurements. The sample was divided also by the IPAQ results (active individuals were considered to be those with ≥150 min/week of physical activity).

Sequential multiple linear regression models were constructed with office BP and ABPM values as dependent variables to adjust for possible confounding variables found on univariate analysis. A sample size of 150 individuals was required to detect a difference of 7 mmHg in systolic BP measured by ABPM [4], considering an alpha error of 5%, a statistical power of 80%, and a SD of 15 mmHg for 24 h systolic BP. *P* values < 0.05 (two tailed) were considered significant. Analyses were performed by SPSS version 18.0 or STATA 12.

## 3. Results and Discussion

### 3.1. Results

A total of 408 people were initially invited, and 257 people were excluded; 155 lived in other cities, 89 refused to participate, 12 dropped out, one died, and one presented a serious medical problem before the beginning of the protocol. Thus, 151 persons with type 2 DM (female, *n* = 97 (64%); white, *n* = 116 (77%)) were included in the study. Mean age was 61.1 ± 8.4 years old, duration of diabetes was 14.3 ± 8.5 years, and BMI was 29.8 ± 4.7 kg/m^2^. Twenty participants (13%) were current smokers, 79 (52%) were living with a partner, and 67 (44%) had completed at least eight years of formal education. Mean HbA1c was 8.5 ± 2.0% (69 mmol/mol). Diabetes was managed with diet alone in 2 participants (1%), oral agents in 74 participants (49%), a combination of oral agents and insulin in 67 participants (45%), and insulin alone in 8 participants (5%). Of the 151 participants evaluated, 139 (92%) had hypertension; six were treated with nonpharmacological therapy alone, and 133 also with antihypertensive agents [(mean of 2.1 ± 1.2 antihypertensive agents per participants; diuretics, *n* = 107 (71%); angiotensin converting enzyme inhibitors, *n* = 91 (60%); *β*-blockers, *n* = 63 (42%); calcium channel blockers, *n* = 45 (30%))]. Mean office BP was 138 ± 18/78 ± 10 mmHg.

The overall mean step count per day was 6391 ± 3357 (median, 5776; interquartile range, 660–19,736), and participants walked a median distance of 3.1 (interquartile range, 2.2–10) km per day. No correlations were observed between step count and office systolic BP (*r* = −0.122, *p* = 0.136) or office diastolic BP (*r* = 0.192, *p* = 0.261). However, significant correlations were found between step count and most ABPM parameters. Inverse correlations between daily mean step count and 24 h systolic BP (*r* = −0.186, *p* = 0.022), 24 h pulse pressure (*r* = −0.210, *p* = 0.010), and 24 h systolic BP load (*r* = −0.177, *p* = 0.030) were detected. There were no associations between step count and 24 h diastolic BP, 24 h mean BP, or 24 h diastolic BP load. Regarding daytime BP measurements, inverse correlations were found between mean step count and daytime systolic BP (*r* = −0.198, *p* = 0.015), daytime pulse pressure (*r* = −0.225, *p* = 0.005), and daytime systolic BP load (*r* = −0.195, *p* = 0.017). No correlations were observed between step count and daytime diastolic BP, daytime mean BP, and daytime diastolic BP load. Nighttime pulse pressure was the only nighttime BP variable correlated with mean daily step counts (*r* = −0.190, *p* = 0.019).

Clinical and laboratory characteristics of participants according to the tertiles of mean step count/day (1st tertile: <4873 steps/day; 2nd tertile: ≥4873 to 7113 steps/day; 3rd tertile: ≥7114 steps/day) are listed in Table 1. Participants in the 1st tertile of daily mean step count were older (*p* = 0.013) than those in the 3rd tertile, and HbA1c was higher in participants in the 2nd tertile than among those in the 3rd tertile (*p* = 0.008). No between-group differences were observed for the other clinical and laboratory parameters.

Participants with the least usual physical activity as evaluated by step count (1st tertile) had higher levels of 24 h systolic BP, 24 h pulse pressure, daytime systolic BP, and daytime pulse pressure than those with higher levels of usual physical activity (2nd and 3rd tertiles) (Table 2). Mean daytime BP and daytime systolic BP load were increased in participants in the 1st tertile as compared with those in the 2nd tertile.

According to self-reported physical activity IPAQ, 136 (90%) participants were classified as active. However, only 18 participants (12%) reported regular planned physical activity in their physical activity log (85% walking, 5% resistance training, 5% biking, and 5% yoga), comprising only 118 minutes of exercise per week on average (range, 35–855 minutes/week).  Table 3 shows the distribution of active individuals, in the different domains of physical activity evaluated by IPAQ, according to daily step count. The percentage of active individuals was similar along the tertiles of daily step count when total physical activity was considered. However, in the domains of transportation, leisure time and a higher frequency of active individuals were found in those participants with higher number of steps/day. Office BP and ABPM values were similar between active and not active individuals classified by the IPAQ domains (data not shown).

Given that BP values were similar among participants in the two upper tertiles of step count and most differences were found on comparisons with the lower tertile, we decided to pool participants belonging to the two upper tertiles for multivariate analysis. Each BP variable was included in a separate linear regression model as the dependent variable, with the dichotomized steps a day (<4873 or ≥4873 steps/day), age, and HbA1c as independent variables. Less than 4873 of mean daily step count was associated with higher office systolic BP (*β* = 6.40, 95% CI 0.31; 12.46, *p* = 0.040), 24 h systolic BP (*β* = 5.32, 95% CI 0.89; 9.74, *p* = 0.019), daytime systolic BP (*β* = 6.29, 95% CI 1.90; 10.69, *p* = 0.005), and daytime mean BP (*β* = 3.24, 95% CI 0.20; 6.28, *p* = 0.037) values, but not with other variables, after adjustments (Table 4).

### 3.2. Discussion

In this sample of people with type 2 DM, low levels of usual physical activity were associated with higher office systolic BP and 24 h and daytime systolic BP, as well as with higher daytime mean BP. These associations remained significant even after adjustment for possible confounding factors. However, no differences in either diastolic or nighttime BP were observed. To the best of our knowledge, the present study is the first to evaluate the association between usual daily physical activity and ABPM profile in participants with type 2 DM.

In our sample, participants walked a mean of 6391 steps/day. This is consistent with previous studies, which have reported mean daily counts of 3448 to 7220 steps in persons with type 2 DM [15, 16, 25]. Use of the pedometer for measurement of daily physical activity has been extensively validated [26–29]. In people with diabetes, step counting correlates with aerobic capacity, as evaluated by VO2max (*r* = 0.43, *p* = 0.02) and with perceived physical fitness (*r* = 0.48, *p* = 0.02) [17]. However, in our analysis, the association between step counts and self-reported levels of overall physical activity, as measured by the IPAQ questionnaire, was quite poor. We found a linear trend between daily step counts and two domains of physical activity (transportation and leisure time), but not for total physical activity. This finding suggests that the IPAQ may not be an appropriate instrument for assessment of usual overall daily physical activity in people with type 2 DM, as it has been demonstrated in other populations [30–32].

There are practical implications of the results presented herein. As previously described, traditionally used cutoff points of steps obtained from pedometers for classification of healthy adults are higher than those observed in this analysis. Thus, the traditional recommendation of walking at least 10,000 steps/day is probably a target difficult to attain for most individuals with type 2 DM [19, 20, 26, 29]. Considering that BP levels of persons with type 2 DM with usual level of daily physical activity in the 2nd tertile (4873–7113 steps/day) were lower than those from the 1st tertile (<4873 steps/day) and similar to those from the 3rd tertile (≥7114 steps/day), we may suggest that people with type 2 DM should be encouraged to walk at least 5000 steps per day. In our opinion, this may be a more feasible target. Indeed, a RCT in patients with type 2 DM and uncontrolled hypertension, we have demonstrated that DASH diet plus walking more than 6000 steps were associated with a clinically relevant reduction in ABPM values [27].

Usually, the cross-sectional design would be a limitation of this kind of study. However, as the information here obtained reflects real-life levels of usual physical exercise and BP profiles, we do not believe that it would be a major drawback. The main limitation of this report is related to an intrinsic characteristic of the device used to evaluate usual physical activity. Pedometers capture only movements of the lower body in the vertical plane and are unable to count other activities such as exercise involving upper limbs, as well as those performed on a bike or inside the water, like swimming.

## 4. Conclusions

In conclusion, people with type 2 DM and low levels of usual physical activity exhibit higher ambulatory BP values in comparison with those who engage in more spontaneous exercise. Prospective cohort studies should be conducted to ascertain whether a causal relationship exists between usual physical activity and ABPM profile.

## Figures and Tables

**Table 1:** Clinical profile of the sample, stratified by step count tertiles.

Variables	Tertiles (steps/day)			*p*
	1st (<4873)	2nd (4873–7113)	3rd (≥7114)	
	*n* = 50	*n* = 50	*n* = 51	
Age (years)	63 ± 8.7	61.6 ± 8.9	58.2 ± 7.3	0.013^∗^
Diabetes duration (years)	13.8 ± 8.5	15.1 ± 8.4	13.5 ± 8.3	0.595
Male sex	15 (30)	19 (38)	20 (39.2)	0.578
White ethnicity	37 (74)	41 (82)	39 (76.5)	0.913
Current smoking	8 (16)	9 (18)	3 (5.9)	0.308
Living with a partner	22 (44)	30 (60)	27 (52.9)	0.506
Education (years)	8.4 ± 4.4	8.8 ± 4.3	9.5 ± 5	0.342
Hypertension	46 (92)	45 (90)	48 (94.1)	0.746
BMI (kg/m^2^)	30.2 ± 4.3	29.7 ± 4.9	29.5 ± 4.7	0.709
Antihypertensive agents	2.1 ± 1.2	1.9 ± 1.2	2.18 ± 1.1	0.435
*Diabetes management*				0.907
Diet alone	0 (0)	1 (2)	1 (2)	
Oral agents	24 (48)	24 (48)	26 (51)	
Insulin alone	4 (8)	2 (4)	2 (3.9)	
Oral agents and insulin	22 (44)	23 (46)	22 (43.1)	
Retinopathy	9 (27)	13 (35)	15 (40)	0.248
Nephropathy	15 (30.1)	20 (42.5)	20 (48.8)	0.171
Cardiovascular disease	8 (17)	5 (10)	6 (12.2)	0.494
Fasting glucose (mg/dL)	177.6 ± 67.5	172.6 ± 87.1	153.2 ± 54	0.190
HbA1c (%) (mmol/mol)	8.6 ± 1.9 (70)	8.9 ± 2.2 (74)	7.8 ± 1.7 (62)	0.008^†^
Total cholesterol (mg/dL)	172.5 ± 38.3	174.3 ± 45.9	176.6 ± 39.8	0.885
LDL cholesterol (mg/dL)	90 ± 37	94 ± 35	96 ± 33	0.423
HDL cholesterol (mg/dL)	40 ± 12	44 ± 13	45 ± 14	0.192
Triglycerides (mg/dL)	171 (106–232)	139 (97–214)	150 (80–228)	0.445
Creatinine (mg/dL)	0.8 ± 0.2	0.9 ± 0.4	0.8 ± 0.2	0.260

^∗^1st versus 3rd tertile; ^†^2nd versus 3rd tertile. Continuous variables are expressed as means ± SD or medians and interquartile ranges, and categorical variables are expressed as absolute (*n*) and relative frequencies (%).

**Table 2:** Blood pressure profiles, stratified by step count tertiles.

Variables	Tertiles (steps/day)			*p*
	1st (<4873)	2nd (4873–7113)	3rd (≥7114)	
	*n* = 50	*n* = 50	*n* = 51	
*Office*				
Systolic BP (mmHg)	142.8 ± 20.7	134.6 ± 14.8	136.3 ± 17.2	0.054
Diastolic BP (mmHg)	78.1 ± 9.9	77.7 ± 9.6	79.5 ± 10.9	0.666
*24 h*				
Systolic BP (mmHg)	133.2 ± 15.4	125.7 ± 11.6	126.8 ± 12.3	0.011^∗^
Diastolic BP (mmHg)	73.7 ± 8.3	72.5 ± 6.5	74.2 ± 8.1	0.510
Mean BP (mmHg)	95.2 ± 9.6	91.3 ± 7	92.8 ± 8.6	0.072
Pulse pressure (mmHg)	58.2 ± 14.9	52.7 ± 12.4	52.5 ± 9.8	0.040^∗^
Systolic BP load (%)	44 (22–76)	26 (8–53)	24 (11–45)	0.014^∗^
Diastolic BP load (%)	8 (3–26)	5 (1–18)	7 (2–27)	0.269
*Daytime*				
Systolic BP (mmHg)	135.9 ± 14.6	127.2 ± 12.4	128.9 ± 11.9	0.003^∗^
Diastolic BP (mmHg)	76.9 ± 8.5	74.6 ± 7.1	77.2 ± 8.4	0.227
Mean BP (mmHg)	98 ± 9.5	93.2 ± 7.8	95.5 ± 8.7	0.025^†^
Pulse pressure (mmHg)	57.7 ± 14.5	52.1 ± 12.6	51.7 ± 9.6	0.028^∗^
Systolic BP load (%)	33 (18–74)	10 (2–31)	20 (7–37)	<0.001^†^
Diastolic BP load (%)	6 (1–29)	2 (0–10)	5 (2–23)	0.022^‡^
*Nighttime*				
Systolic BP (mmHg)	127.6 ± 18.7	121.4 ± 13.4	121.8 ± 15.3	0.096
Diastolic BP (mmHg)	67.2 ± 9.6	67 ± 7.9	67 ± 12	0.993
Mean BP (mmHg)	89.3 ± 11.8	86.4 ± 9.1	85.9 ± 14.6	0.307
Pulse pressure (mmHg)	59.3 ± 15.8	54.4 ± 10.1	53.5 ± 12.5	0.061
Systolic BP load (%)	54 (24–92)	52 (11–80)	41 (15–82)	0.424
Diastolic BP load (%)	5 (0–26)	5 (0–14)	8 (0–24)	0.416

^∗^1st versus 2nd and 3rd tertiles; ^†^1st versus 2nd tertile; ^‡^2nd versus 3rd tertile. Variables are expressed as means ± SD or median (interquartile range).

**Table 3:** Frequency of active individuals based on IPAQ results (≥150 min/week) stratified by step count tertiles.

Domains of physical activity	Tertiles (steps/day)			*p* ^∗^
	1st (<4873)	2nd (4873–7113)	3rd (≥7114)	
	*n* = 50	*n* = 50	*n* = 51	
Occupational	12 (24)	11 (22)	20 (39)	0.090
Transportation	13 (26)	17 (34)	24 (47)	0.028
Household	33 (66)	32 (64)	30 (59)	0.456
Leisure time	9 (18)	11 (22)	19 (37)	0.027
Transportation + leisure time	23 (46)	25 (50)	32 (63)	0.092
Total walking	27 (54)	24 (48)	36 (71)	0.091
Total physical activity	44 (88)	43 (86)	49 (96)	0.174

^∗^Chi-square test for linear trends. Variables are expressed as absolute (*n*) and relative frequencies (%).

**Table 4:** Linear regression between steps count per day (lower tertile versus the two upper tertiles) and blood pressure profile.

Variables	*β*_unadjusted_ (95% CI)	*p*	*β*_adjusted_ (95% CI)	*p*
*Office*				
Systolic BP (mmHg)	7.79 (1.74; 13.84)	0.012	6.40 (0.31; 12.46)	0.040
Diastolic BP (mmHg)	−0.65 (−4.06; 2.75)	0.705	0.43 (−2.88; 3.74)	0.798
*24 h*				
Systolic BP (mmHg)	6.96 (−2.44; −11.48)	0.003	5.32 (0.89; 9.74)	0.019
Diastolic BP (mmHg)	0.38 (−2.26; 3.03)	0.775	0.69 (−1.98; 3.37)	0.609
Mean BP (mmHg)	3.16 (0.25; 6.07)	0.033	2.63 (−0.31; 5.58)	0.079
Pulse pressure (mmHg)	5.59 (1.30; 9.88)	0.011	3.41 (−0.60; 7.41)	0.095
*Daytime*				
Systolic BP (mmHg)	7.80 (3.34; 12.26)	0.001	6.29 (1.90; 10.69)	0.005
Diastolic BP (mmHg)	0.92 (−1.86; 3.70)	0.514	1.30 (−1.50; 4.11)	0.360
Mean BP (mmHg)	3.65 (0.66; 6.64)	0.017	3.24 (0.20; 6.28)	0.037
Pulse pressure (mmHg)	5.81 (1.58; 10.04)	0.007	3.71 (−0.26; 7.68)	0.067
*Nighttime*				
Systolic BP (mmHg)	6.00 (0.58; 11.04)	0.030	4.06 (−1.25; 9.38)	0.133
Diastolic BP (mmHg)	0.21 (−3.22; 3.64)	0.904	0.55 (−2.94; 4.05)	0.755
Mean BP (mmHg)	3.20 (−0.92; 7.32)	0.127	2.63 (−1.57; 6.83)	0.218
Pulse pressure (mmHg)	5.31 (0.88; 9.74)	0.019	3.12 (−1.04; 7.30)	0.141

Adjusted for age and HbA1c.
